# 1-Di­chloro­acetyl-*t*-3-isopropyl-*r*-2,*c*-6-di­phenyl­piperidin-4-one

**DOI:** 10.1107/S1600536813019582

**Published:** 2013-07-27

**Authors:** P. Sugumar, R. Kayalvizhi, R. Mini, S. Ponnuswamy, M. N. Ponnuswamy

**Affiliations:** a Centre of Advanced Study in Crystallography and Biophysics, University of Madras, Guindy Campus, Chennai 600 025, India; b Department of Chemistry, Government Arts College (Autonomous), Coimbatore 641 018, India

## Abstract

In the title compound, C_22_H_23_Cl_2_NO_2_, the piperidine ring adopts a twist-boat conformation. The phenyl rings substituted at the 2- and 6-positions of the piperidine ring subtend dihedral angles of 60.6 (2) and 84.2 (1)°, respectively, with the mean plane of the piperidine ring. In the crystal, mol­ecules are linked by C—H⋯O inter­actions into zigzag chains running along the *c-*axis direction.

## Related literature

For the biological activity of piperidine derivatives, see: Aridoss *et al.* (2009 ▶); Nalanishi *et al.* (1974 ▶); Michael (2001 ▶); Pinder (1992 ▶); Rubiralta *et al.* (1991 ▶). For puckering parameters, see: Cremer & Pople (1975 ▶). For asymmetry parameters, see: Nardelli (1983 ▶).
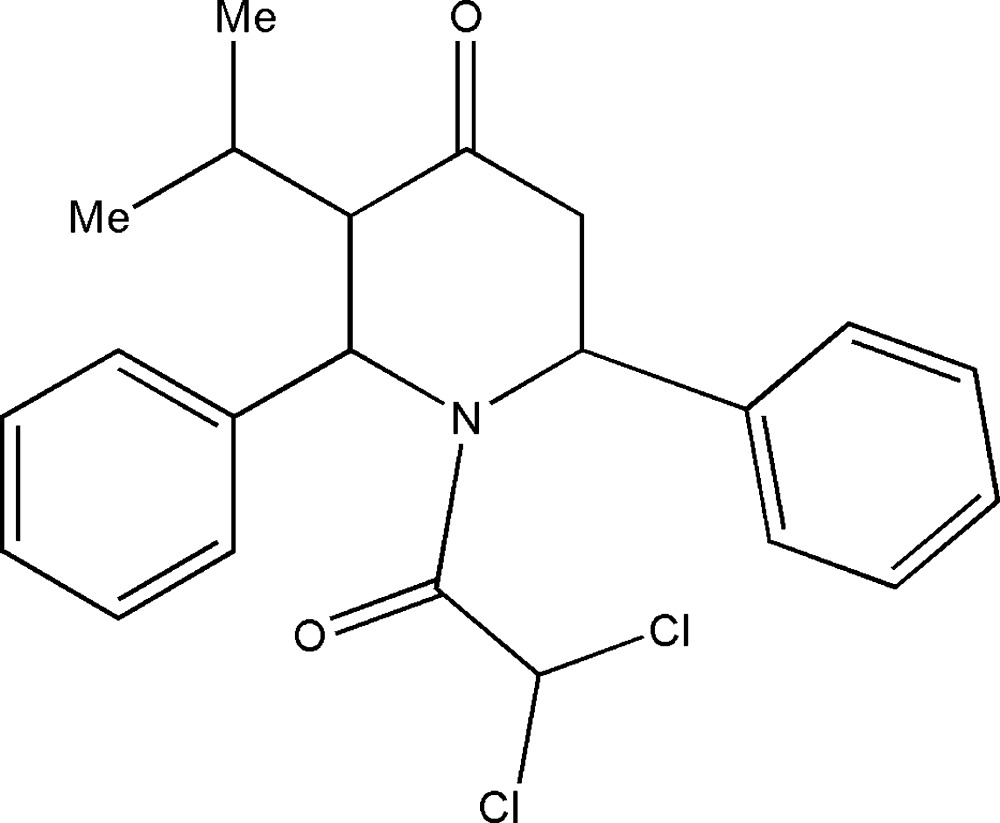


## Experimental

### 

#### Crystal data

C_22_H_23_Cl_2_NO_2_
*M* *_r_* = 404.31
Orthorhombic, 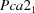

*a* = 18.4336 (14) Å
*b* = 9.4516 (7) Å
*c* = 11.7077 (9) Å
*V* = 2039.8 (3) Å^3^
*Z* = 4
Mo *K*α radiation
μ = 0.34 mm^−1^
*T* = 293 K
0.22 × 0.20 × 0.18 mm

#### Data collection

Bruker SMART APEXII CCD diffractometer
Absorption correction: multi-scan (*SADABS*; Bruker, 2008 ▶) *T*_min_ = 0.929, *T*_max_ = 0.941
10509 measured reflections
4437 independent reflections
3882 reflections with *I* > 2σ(*I*)
*R*_int_ = 0.022

#### Refinement

*R*[*F* ^2^ > 2σ(*F* ^2^)] = 0.048
*wR*(*F* ^2^) = 0.136
*S* = 1.04
4437 reflections
244 parameters
1 restraint
H-atom parameters constrained
Δρ_max_ = 0.69 e Å^−3^
Δρ_min_ = −0.47 e Å^−3^
Absolute structure: Flack (1983 ▶), 1759 Friedel pairs
Absolute structure parameter: −0.08 (8)

### 

Data collection: *APEX2* (Bruker, 2008 ▶); cell refinement: *SAINT* (Bruker, 2008 ▶); data reduction: *SAINT*; program(s) used to solve structure: *SHELXS97* (Sheldrick, 2008 ▶); program(s) used to refine structure: *SHELXL97* (Sheldrick, 2008 ▶); molecular graphics: *ORTEP-3 for Windows* (Farrugia, 2012 ▶); software used to prepare material for publication: *SHELXL97* and *PLATON* (Spek, 2009 ▶).

## Supplementary Material

Crystal structure: contains datablock(s) global, I. DOI: 10.1107/S1600536813019582/bt6918sup1.cif

Structure factors: contains datablock(s) I. DOI: 10.1107/S1600536813019582/bt6918Isup2.hkl

Supplementary material file. DOI: 10.1107/S1600536813019582/bt6918Isup3.cml

Additional supplementary materials:  crystallographic information; 3D view; checkCIF report

## Figures and Tables

**Table 1:** Hydrogen-bond geometry (Å, °)

*D*—H⋯*A*	*D*—H	H⋯*A*	*D*⋯*A*	*D*—H⋯*A*
C2—H2⋯O1^i^	0.98	2.39	3.144 (3)	133

Symmetry code: (i) 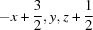.

**Fig. 1.**
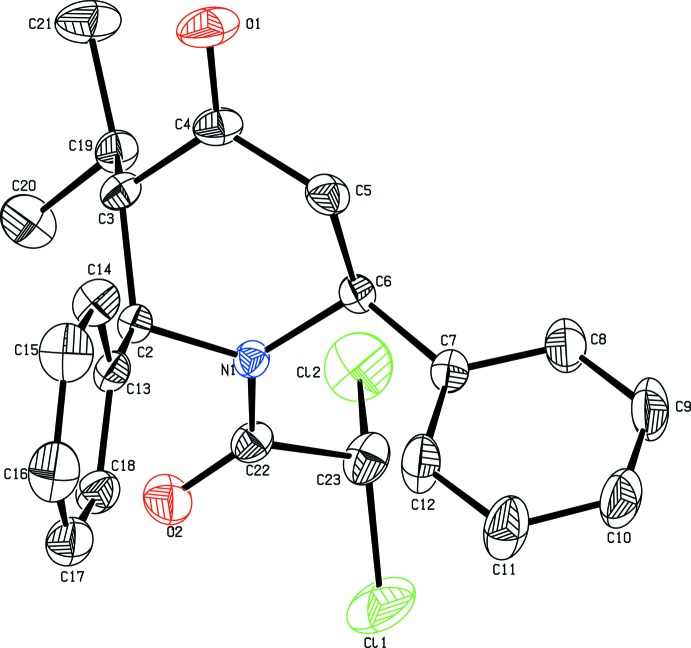
The molecular structure of the title compound, showing the atomic numbering and displacement ellipsoids drawn at 50% probability level.

**Fig. 2.**
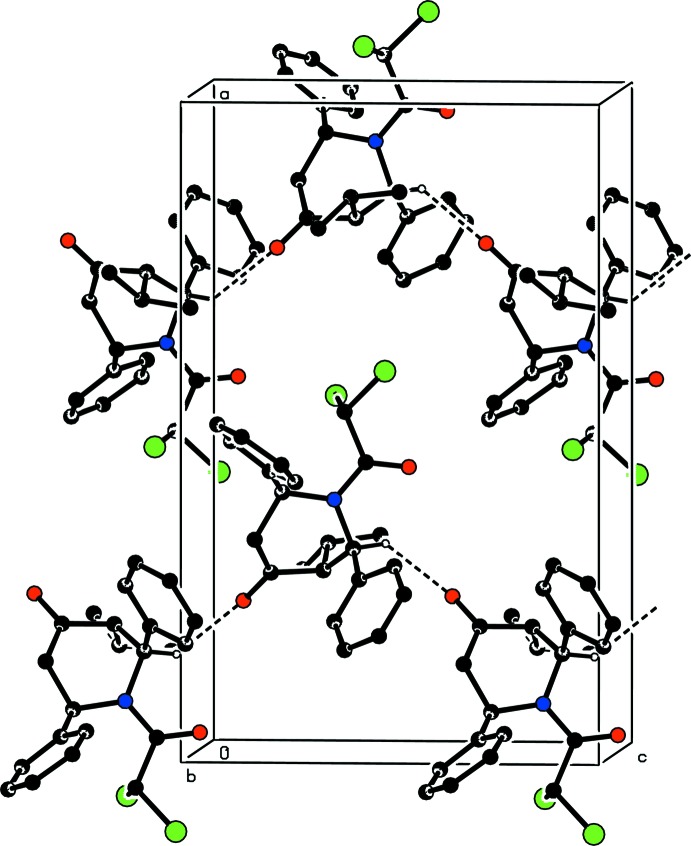
The crystal packing of the molecules. H atoms not involved in hydrogen bonding (dashed lines) have been omitted for clarity.

## supplementary crystallographic information

### Comment

Piperidine derivatives are valuable heterocyclic compounds in the field of medicinal chemistry. The compounds possessing an amide bond linkage have a wide range of biological activities such as antimicrobial, anti-inflammatory, antiviral, antimalarial and general anesthetics (Aridoss *et al.*, 2009). Functionalized piperidines are familiar substructures found in biologically active natural products and synthetic pharmaceuticals (Michael, 2001; Pinder, 1992; Rubiralta *et al.*, 1991). Piperidines have been found to exhibit blood cholesterol-lowering activities (Nalanishi *et al.*, 1974). Against this background and to ascertain the molecular structure and conformation, the X-ray crystal structure determination of the title compound has been carried out.

The *ORTEP* plot of the molecule is shown in Fig. 1. The piperidine ring adopts a twist-boat conformation, with puckering parameters (Cremer & Pople, 1975) and asymmetry parameters (Nardelli, 1983) of: q_2_ = 0.632 (2) Å, q_3_ = -0.088 (3) Å, φ_2_ = 254.1 (2)° and Δ_s_(N1 and C4) = 69.8 (2)°.

The phenyl rings at the 2- and 6-positions of the piperidine ring occupy axial and equatorial orientation, as evidenced from the torsion angles C4—C3—C2—C13 = 70.6 (3)° and C4—C5—C6—C7 = -165.0 (2)°, respectively. The two phenyl rings are approximately perpendicular to each other with a dihedral angle of 86.6 (2)°. The best plane of the piperidine ring subtends angles of 60.6 (2)° and 84.2 (1)° with the attached phenyl rings [C7—C12 and C13—C18].

The carbonyl group is oriented *syn-periplanar* to C2 [C2—N1—C22—O2 = -10.9 (4)°] and *anti-periplanar* to C6 [C6—N1—C22—O2 = 178.2 (2)°].

The crystal packing reveals that the symmetry-related molecules are linked through a network of C—H···O type of intermolecular interactions. Atom C2 (*x*, *y*, *z*) donates a proton to atom O1 (-*x* + 3/2, *y*, *z* + 1/2), which form a chain in *zigzag* fashion running along the *c* direction as shown in Fig. 2.

### Experimental

t-3-Isopropyl-r-2,c-6-diphenylpiperidin-4-ones (5 mmol) was dissolved in 60 ml of anhydrous benzene. To this solution, dichloroacetylchloride (20 mmol) and triethylamine (20 mmol) were added and the reaction mixture was allowed to stir for 8 h. The course of the reaction was monitored by TLC. The organic layer was dried over anhydrous Na_2_SO_4_ and the resulting pasty mass was purified by recrystallization from ethyl acetate. Yield: 70%, m.p. 156–158°C.

### Refinement

N and C-bound H atoms were positioned geometrically (C—H = 0.93–0.98 Å) and allowed to ride on their parent atoms, with *U*_iso_(H) = 1.5*U*_eq_(C) for methyl H atoms and 1.2*U*_eq_(C) for all other H atoms.

### Crystal data

**Table:**

C_22_H_23_Cl_2_NO_2_	*F*(000) = 848
*M**_r_* = 404.31	*D*_x_ = 1.317 Mg m^−^^3^
Orthorhombic, *P**c**a*2_1_	Mo *K*α radiation, λ = 0.71073 Å
Hall symbol: P 2c -2ac	Cell parameters from 3882 reflections
*a* = 18.4336 (14) Å	θ = 2.2–28.4°
*b* = 9.4516 (7) Å	µ = 0.34 mm^−^^1^
*c* = 11.7077 (9) Å	*T* = 293 K
*V* = 2039.8 (3) Å^3^	Block, yellow
*Z* = 4	0.22 × 0.20 × 0.18 mm

### Data collection

**Table:**

Bruker SMART APEXII CCD diffractometer	4437 independent reflections
Radiation source: fine-focus sealed tube	3882 reflections with *I* > 2σ(*I*)
Graphite monochromator	*R*_int_ = 0.022
ω and φ scans	θ_max_ = 28.4°, θ_min_ = 2.2°
Absorption correction: multi-scan (*SADABS*; Bruker, 2008)	*h* = −14→24
*T*_min_ = 0.929, *T*_max_ = 0.941	*k* = −12→12
10509 measured reflections	*l* = −11→15

### Refinement

**Table:**

Refinement on *F*^2^	Secondary atom site location: difference Fourier map
Least-squares matrix: full	Hydrogen site location: inferred from neighbouring sites
*R*[*F*^2^ > 2σ(*F*^2^)] = 0.048	H-atom parameters constrained
*wR*(*F*^2^) = 0.136	*w* = 1/[σ^2^(*F*_o_^2^) + (0.0726*P*)^2^ + 0.6068*P*] where *P* = (*F*_o_^2^ + 2*F*_c_^2^)/3
*S* = 1.04	(Δ/σ)_max_ = 0.001
4437 reflections	Δρ_max_ = 0.69 e Å^−^^3^
244 parameters	Δρ_min_ = −0.47 e Å^−^^3^
1 restraint	Absolute structure: Flack (1983), 1759 Friedel pairs
Primary atom site location: structure-invariant direct methods	Flack parameter: −0.08 (8)

### Special details

**Table:**

Geometry. All esds (except the esd in the dihedral angle between two l.s. planes) are estimated using the full covariance matrix. The cell esds are taken into account individually in the estimation of esds in distances, angles and torsion angles; correlations between esds in cell parameters are only used when they are defined by crystal symmetry. An approximate (isotropic) treatment of cell esds is used for estimating esds involving l.s. planes.
Refinement. Refinement of F^2^ against ALL reflections. The weighted R-factor wR and goodness of fit S are based on F^2^, conventional R-factors R are based on F, with F set to zero for negative F^2^. The threshold expression of F^2^ > 2sigma(F^2^) is used only for calculating R-factors(gt) etc. and is not relevant to the choice of reflections for refinement. R-factors based on F^2^ are statistically about twice as large as those based on F, and R- factors based on ALL data will be even larger.

### Fractional atomic coordinates and isotropic or equivalent isotropic displacement parameters (Å^2^)

**Table:**

	*x*	*y*	*z*	*U*_iso_*/*U*_eq_	
C2	0.67099 (11)	0.9032 (2)	0.91976 (19)	0.0306 (4)	
H2	0.6651	0.9516	0.9932	0.037*	
C3	0.70822 (12)	1.0091 (2)	0.8399 (2)	0.0352 (4)	
H3	0.7584	1.0209	0.8659	0.042*	
C4	0.71030 (13)	0.9535 (3)	0.7191 (2)	0.0421 (5)	
C5	0.65663 (14)	0.8387 (2)	0.6894 (2)	0.0414 (5)	
H5A	0.6797	0.7479	0.7023	0.050*	
H5B	0.6459	0.8452	0.6084	0.050*	
C6	0.58452 (12)	0.8409 (2)	0.7551 (2)	0.0333 (4)	
H6	0.5536	0.9146	0.7221	0.040*	
C7	0.54776 (12)	0.6975 (2)	0.7366 (2)	0.0381 (5)	
C8	0.5026 (2)	0.6797 (4)	0.6451 (4)	0.0778 (12)	
H8	0.4919	0.7560	0.5979	0.093*	
C9	0.4727 (3)	0.5481 (5)	0.6228 (4)	0.0948 (16)	
H9	0.4419	0.5374	0.5604	0.114*	
C10	0.4871 (2)	0.4346 (4)	0.6897 (3)	0.0685 (9)	
H10	0.4683	0.3460	0.6720	0.082*	
C11	0.5299 (2)	0.4531 (3)	0.7837 (4)	0.0712 (11)	
H11	0.5386	0.3775	0.8327	0.085*	
C12	0.56030 (17)	0.5841 (3)	0.8064 (3)	0.0601 (9)	
H12	0.5898	0.5951	0.8703	0.072*	
C13	0.71354 (11)	0.7680 (2)	0.9446 (2)	0.0348 (5)	
C14	0.77614 (13)	0.7293 (3)	0.8883 (3)	0.0464 (6)	
H14	0.7927	0.7833	0.8272	0.056*	
C15	0.81513 (18)	0.6090 (3)	0.9224 (3)	0.0604 (8)	
H15	0.8577	0.5846	0.8845	0.072*	
C16	0.79074 (18)	0.5271 (3)	1.0114 (3)	0.0623 (8)	
H16	0.8163	0.4467	1.0332	0.075*	
C17	0.72847 (17)	0.5646 (3)	1.0682 (3)	0.0567 (7)	
H17	0.7120	0.5096	1.1287	0.068*	
C18	0.69012 (14)	0.6841 (3)	1.0356 (2)	0.0442 (6)	
H18	0.6482	0.7088	1.0750	0.053*	
C19	0.67141 (14)	1.1579 (2)	0.8402 (2)	0.0400 (5)	
H19	0.6234	1.1489	0.8048	0.048*	
C20	0.6614 (2)	1.2139 (3)	0.9603 (3)	0.0606 (8)	
H20A	0.6331	1.1479	1.0039	0.091*	
H20B	0.6368	1.3033	0.9575	0.091*	
H20C	0.7080	1.2259	0.9958	0.091*	
C21	0.7155 (2)	1.2637 (3)	0.7707 (3)	0.0675 (9)	
H21A	0.7222	1.2280	0.6946	0.101*	
H21B	0.7620	1.2774	0.8061	0.101*	
H21C	0.6902	1.3523	0.7675	0.101*	
C22	0.54042 (12)	0.8987 (2)	0.9506 (2)	0.0383 (5)	
C23	0.46416 (13)	0.9023 (3)	0.8997 (3)	0.0506 (7)	
H23	0.4622	0.8437	0.8307	0.061*	
N1	0.59629 (9)	0.87405 (18)	0.87687 (17)	0.0309 (4)	
O1	0.75440 (14)	0.9925 (2)	0.6505 (2)	0.0671 (6)	
O2	0.54794 (11)	0.9232 (3)	1.05165 (19)	0.0602 (6)	
Cl2	0.44564 (6)	1.08090 (11)	0.86504 (12)	0.0926 (4)	
Cl1	0.40029 (5)	0.84201 (14)	1.00044 (13)	0.0960 (4)	

### Atomic displacement parameters (Å^2^)

**Table:**

	*U*^11^	*U*^22^	*U*^33^	*U*^12^	*U*^13^	*U*^23^
C2	0.0295 (9)	0.0290 (9)	0.0334 (11)	−0.0014 (7)	−0.0020 (7)	−0.0024 (8)
C3	0.0349 (9)	0.0292 (9)	0.0414 (12)	−0.0046 (7)	0.0037 (8)	−0.0004 (9)
C4	0.0450 (11)	0.0359 (11)	0.0454 (14)	0.0003 (9)	0.0145 (10)	0.0012 (10)
C5	0.0476 (12)	0.0440 (12)	0.0325 (12)	0.0004 (10)	0.0069 (9)	−0.0047 (10)
C6	0.0358 (9)	0.0345 (9)	0.0294 (11)	0.0002 (8)	−0.0024 (8)	−0.0015 (9)
C7	0.0363 (10)	0.0391 (11)	0.0389 (13)	−0.0020 (8)	−0.0029 (9)	−0.0037 (9)
C8	0.101 (3)	0.0673 (19)	0.065 (2)	−0.0354 (19)	−0.040 (2)	0.0188 (17)
C9	0.129 (4)	0.082 (2)	0.073 (3)	−0.051 (2)	−0.055 (3)	0.008 (2)
C10	0.077 (2)	0.0510 (15)	0.078 (2)	−0.0206 (15)	−0.0209 (18)	−0.0113 (15)
C11	0.081 (2)	0.0369 (13)	0.096 (3)	−0.0077 (13)	−0.037 (2)	0.0021 (15)
C12	0.0698 (18)	0.0399 (13)	0.070 (2)	−0.0064 (12)	−0.0348 (16)	0.0017 (13)
C13	0.0317 (9)	0.0298 (9)	0.0428 (13)	−0.0032 (7)	−0.0055 (9)	−0.0002 (9)
C14	0.0407 (11)	0.0413 (12)	0.0572 (17)	0.0038 (9)	0.0013 (11)	0.0002 (12)
C15	0.0531 (15)	0.0546 (15)	0.073 (2)	0.0193 (13)	−0.0017 (14)	−0.0057 (15)
C16	0.0674 (18)	0.0362 (12)	0.083 (2)	0.0118 (12)	−0.0197 (17)	0.0005 (14)
C17	0.0659 (17)	0.0392 (13)	0.0649 (19)	−0.0080 (11)	−0.0152 (14)	0.0113 (13)
C18	0.0427 (11)	0.0381 (11)	0.0517 (16)	−0.0034 (9)	−0.0032 (11)	0.0042 (11)
C19	0.0457 (12)	0.0291 (10)	0.0451 (14)	−0.0003 (8)	−0.0019 (10)	0.0012 (9)
C20	0.094 (2)	0.0332 (12)	0.0545 (18)	0.0094 (13)	0.0004 (16)	−0.0070 (12)
C21	0.094 (2)	0.0342 (13)	0.074 (2)	−0.0049 (13)	0.0195 (19)	0.0127 (14)
C22	0.0305 (10)	0.0411 (11)	0.0433 (14)	−0.0007 (8)	0.0053 (9)	−0.0033 (10)
C23	0.0322 (11)	0.0550 (14)	0.065 (2)	0.0027 (10)	0.0052 (11)	−0.0120 (13)
N1	0.0275 (7)	0.0332 (8)	0.0319 (10)	−0.0009 (6)	0.0007 (7)	−0.0015 (7)
O1	0.0794 (14)	0.0592 (11)	0.0628 (14)	−0.0189 (11)	0.0378 (12)	−0.0073 (10)
O2	0.0470 (10)	0.0915 (16)	0.0422 (12)	−0.0024 (10)	0.0119 (8)	−0.0122 (10)
Cl2	0.0772 (6)	0.0778 (6)	0.1229 (10)	0.0262 (5)	−0.0136 (6)	0.0178 (6)
Cl1	0.0470 (4)	0.1175 (8)	0.1233 (10)	−0.0192 (5)	0.0235 (5)	0.0129 (7)

### Geometric parameters (Å, º)

**Table:**

C2—N1	1.491 (2)	C13—C14	1.378 (3)
C2—C13	1.527 (3)	C13—C18	1.396 (4)
C2—C3	1.532 (3)	C14—C15	1.403 (4)
C2—H2	0.9800	C14—H14	0.9300
C3—C4	1.509 (4)	C15—C16	1.374 (5)
C3—C19	1.562 (3)	C15—H15	0.9300
C3—H3	0.9800	C16—C17	1.373 (5)
C4—O1	1.200 (3)	C16—H16	0.9300
C4—C5	1.509 (3)	C17—C18	1.386 (4)
C5—C6	1.536 (3)	C17—H17	0.9300
C5—H5A	0.9700	C18—H18	0.9300
C5—H5B	0.9700	C19—C20	1.514 (4)
C6—N1	1.476 (3)	C19—C21	1.524 (4)
C6—C7	1.531 (3)	C19—H19	0.9800
C6—H6	0.9800	C20—H20A	0.9600
C7—C8	1.367 (4)	C20—H20B	0.9600
C7—C12	1.368 (4)	C20—H20C	0.9600
C8—C9	1.386 (5)	C21—H21A	0.9600
C8—H8	0.9300	C21—H21B	0.9600
C9—C10	1.354 (6)	C21—H21C	0.9600
C9—H9	0.9300	C22—O2	1.213 (3)
C10—C11	1.365 (5)	C22—N1	1.364 (3)
C10—H10	0.9300	C22—C23	1.527 (3)
C11—C12	1.385 (4)	C23—Cl1	1.762 (3)
C11—H11	0.9300	C23—Cl2	1.769 (3)
C12—H12	0.9300	C23—H23	0.9800
			
N1—C2—C13	112.56 (16)	C18—C13—C2	117.5 (2)
N1—C2—C3	109.19 (18)	C13—C14—C15	120.5 (3)
C13—C2—C3	115.65 (18)	C13—C14—H14	119.7
N1—C2—H2	106.3	C15—C14—H14	119.7
C13—C2—H2	106.3	C16—C15—C14	120.3 (3)
C3—C2—H2	106.3	C16—C15—H15	119.8
C4—C3—C2	110.86 (18)	C14—C15—H15	119.8
C4—C3—C19	109.1 (2)	C17—C16—C15	119.7 (3)
C2—C3—C19	113.09 (18)	C17—C16—H16	120.2
C4—C3—H3	107.9	C15—C16—H16	120.2
C2—C3—H3	107.9	C16—C17—C18	120.3 (3)
C19—C3—H3	107.9	C16—C17—H17	119.9
O1—C4—C3	122.5 (2)	C18—C17—H17	119.9
O1—C4—C5	120.7 (2)	C17—C18—C13	121.0 (3)
C3—C4—C5	116.7 (2)	C17—C18—H18	119.5
C4—C5—C6	116.24 (19)	C13—C18—H18	119.5
C4—C5—H5A	108.2	C20—C19—C21	109.4 (2)
C6—C5—H5A	108.2	C20—C19—C3	111.7 (2)
C4—C5—H5B	108.2	C21—C19—C3	111.0 (2)
C6—C5—H5B	108.2	C20—C19—H19	108.2
H5A—C5—H5B	107.4	C21—C19—H19	108.2
N1—C6—C7	112.94 (19)	C3—C19—H19	108.2
N1—C6—C5	111.08 (18)	C19—C20—H20A	109.5
C7—C6—C5	107.48 (18)	C19—C20—H20B	109.5
N1—C6—H6	108.4	H20A—C20—H20B	109.5
C7—C6—H6	108.4	C19—C20—H20C	109.5
C5—C6—H6	108.4	H20A—C20—H20C	109.5
C8—C7—C12	118.4 (2)	H20B—C20—H20C	109.5
C8—C7—C6	119.3 (2)	C19—C21—H21A	109.5
C12—C7—C6	122.3 (2)	C19—C21—H21B	109.5
C7—C8—C9	120.0 (3)	H21A—C21—H21B	109.5
C7—C8—H8	120.0	C19—C21—H21C	109.5
C9—C8—H8	120.0	H21A—C21—H21C	109.5
C10—C9—C8	121.6 (3)	H21B—C21—H21C	109.5
C10—C9—H9	119.2	O2—C22—N1	124.3 (2)
C8—C9—H9	119.2	O2—C22—C23	118.8 (2)
C9—C10—C11	118.6 (3)	N1—C22—C23	116.9 (2)
C9—C10—H10	120.7	C22—C23—Cl1	110.3 (2)
C11—C10—H10	120.7	C22—C23—Cl2	106.77 (18)
C10—C11—C12	120.2 (3)	Cl1—C23—Cl2	109.47 (15)
C10—C11—H11	119.9	C22—C23—H23	110.1
C12—C11—H11	119.9	Cl1—C23—H23	110.1
C7—C12—C11	121.1 (3)	Cl2—C23—H23	110.1
C7—C12—H12	119.4	C22—N1—C6	122.46 (18)
C11—C12—H12	119.4	C22—N1—C2	116.91 (19)
C14—C13—C18	118.2 (2)	C6—N1—C2	120.02 (17)
C14—C13—C2	124.1 (2)		
			
N1—C2—C3—C4	−57.6 (2)	C18—C13—C14—C15	−0.3 (4)
C13—C2—C3—C4	70.6 (2)	C2—C13—C14—C15	174.9 (3)
N1—C2—C3—C19	65.3 (2)	C13—C14—C15—C16	0.9 (5)
C13—C2—C3—C19	−166.52 (19)	C14—C15—C16—C17	−0.9 (5)
C2—C3—C4—O1	−156.1 (3)	C15—C16—C17—C18	0.3 (5)
C19—C3—C4—O1	78.7 (3)	C16—C17—C18—C13	0.4 (4)
C2—C3—C4—C5	20.3 (3)	C14—C13—C18—C17	−0.4 (4)
C19—C3—C4—C5	−104.8 (2)	C2—C13—C18—C17	−175.9 (2)
O1—C4—C5—C6	−153.7 (3)	C4—C3—C19—C20	174.8 (2)
C3—C4—C5—C6	29.8 (3)	C2—C3—C19—C20	50.9 (3)
C4—C5—C6—N1	−41.1 (3)	C4—C3—C19—C21	−62.9 (3)
C4—C5—C6—C7	−165.1 (2)	C2—C3—C19—C21	173.3 (2)
N1—C6—C7—C8	148.1 (3)	O2—C22—C23—Cl1	−34.0 (3)
C5—C6—C7—C8	−89.1 (3)	N1—C22—C23—Cl1	148.94 (19)
N1—C6—C7—C12	−34.1 (3)	O2—C22—C23—Cl2	84.8 (3)
C5—C6—C7—C12	88.7 (3)	N1—C22—C23—Cl2	−92.2 (2)
C12—C7—C8—C9	−2.2 (6)	O2—C22—N1—C6	178.3 (2)
C6—C7—C8—C9	175.7 (4)	C23—C22—N1—C6	−4.8 (3)
C7—C8—C9—C10	−0.1 (8)	O2—C22—N1—C2	−10.7 (4)
C8—C9—C10—C11	2.7 (8)	C23—C22—N1—C2	166.21 (19)
C9—C10—C11—C12	−2.9 (7)	C7—C6—N1—C22	−67.1 (3)
C8—C7—C12—C11	1.9 (5)	C5—C6—N1—C22	172.1 (2)
C6—C7—C12—C11	−175.9 (3)	C7—C6—N1—C2	122.20 (19)
C10—C11—C12—C7	0.7 (6)	C5—C6—N1—C2	1.4 (3)
N1—C2—C13—C14	117.0 (2)	C13—C2—N1—C22	106.7 (2)
C3—C2—C13—C14	−9.4 (3)	C3—C2—N1—C22	−123.5 (2)
N1—C2—C13—C18	−67.7 (3)	C13—C2—N1—C6	−82.1 (2)
C3—C2—C13—C18	165.8 (2)	C3—C2—N1—C6	47.8 (2)

### Hydrogen-bond geometry (Å, º)

**Table:**

*D*—H···*A*	*D*—H	H···*A*	*D*···*A*	*D*—H···*A*
C2—H2···O1^i^	0.98	2.39	3.144 (3)	133

Symmetry code: (i) −*x*+3/2, *y*, *z*+1/2.
